# Intra-mural Fibroid Tumor

**Published:** 1877-08

**Authors:** Mary H. Thompson


### INTRA-MURAL FIBROID TUMOR.

BY DR. MARY H. THOMPSON.

Mrs. M. E. H., aged 32; American. She was a widow with one child, a boy about eight years of age; entered the hospital Jan. 5, 1876.

Said she had been sick since July, '75, with catarrh of the womb, and had been treated for the same. In December of the same year, while menstruating, was taken very sick; thought she "took cold."

She is rather pale, with tongue red and glazed; slight fever; great pain in the abdomen and lower part of back; has slight metrorrhœa. The abdomen slightly dull on percussion at the level of umbilicus, increasing in dullness to pubis.

Examination per vaginam reveals a disk of inflamed cellular tissue, extending across the pelvis at the junction of the vaginal and supra-vaginal portion of the cervix uteri, and holding that organ and roof of vagina quite immovable, obscuring to the touch the outlines of the corpus uteri.

The appetite is variable; rarely any headache; habitual constipation; very little difficulty in micturition at any time.

Feb. 13. Cellulitis rapidly disappearing; some pus evacuated from the rectum.

Feb. 14. Prof. Byford is called in consultation. The cellular tissue is found sufficiently softened to enable us to map out the outlines of the corpus uteri, which is enlarged to the size of a large orange, and of a globular shape, and thought to contain an intra-mural fibroid tumor, occupying the anterior wall of the body.

The patient continued improving for about three months, taking from 20 to 30 drops of Squibb's fluid extract of ergot a day, but with little or no effect on the tumor. She was then dismissed to obtain a change of air. While out she visited my office occasionally, that I might watch her condition, and that she might be readmitted when necessary. But as the hospital was crowded, I was obliged to refuse her admittance. Yet while traveling about, her metrorrhagia returned more than previously.

Dec. 19. She came into the hospital again. Said that during her absence she had been in another hospital, and that a sponge tent had been used, which was thought to have cured her, but at this time she was thin and bloodless.

The tumor was apparently increasing. From this time, whenever an examination was made per vaginam, a slight hæmorrhage was caused, especially if the finger was introduced, however carefully, into the uterus. The lining of the cervix felt slightly rough as far as could be reached, which was $\frac{5}{8}$ of an inch in extent from the os. Menstruation would come on like a sudden hæmorrhage, frequently ceasing in a day, and as suddenly as it began. On several occasions she became pulseless, and was not expected to survive. At these times Gallic acid was the only drug which seemed to have any effect upon the hæmorrhage.

Jan. 8. The pain was gradually increasing, and could be controlled only by opiates.

Jan. 27. Diarrhœa came on, succeeding constipation; these conditions ever afterwards alternating. Dysenteric pains accompanying the former, and a "continued grinding pain" the latter.

May 8. Is in great pain, unless under the influence of morphine.

May 14. Is but a skeleton; feet swelling; tongue red, dry, and glazed; taste unnatural; complete anorexia; is in great pain; urine thick and scant; much exhausted and failing.

May 17. Dimness of sight in left eye, which continues about three days; is the only paralysis of the nerves of sensation which occurred.

June 1. Death occurred at 11 P. M.

Post-mortem examination fourteen hours after death.

Body much emaciated. Adipose tissue nearly all absent, muscles shrivelled. Small tumors of lymphatic glands (?) about the size and shape of an apple-seed were found on the abdomen, beneath the subcutaneous tissue, which would roll under the finger from side to side. The lymphatics in the vaginal region were greatly enlarged.

The foot, left leg and thigh were greatly œdematous. The abdomen was greatly distended and tympanitic.

The abdomen and pelvis only were examined. When the first incision was made through the abdominal wall, the intestines formed large hernial protrusions on account of their distension with gas and half liquid fæces, and their walls were so fragile as to rupture while lying exposed to the atmosphere.

The womb was not found so large by several times its size as was expected; it was externally of nearly its normal shape; but in its width, which was about four inches, including, on one side, an apparent fallopian tube, twisted about so that its os abdominalis was adherent, and incorporated with the uterine body; on the opposite side are apparent enlarged ovaries, which had been adherent to and formed a part of the mass which included the womb.

It appeared like a fibroid, degenerated into a friable mass of a dark brown or brownish red color, yet containing nodules of hard white fibroid material, intermixed with the dark mass.

The pelvic connective tissue was changed into the same material, only the fibroid nodules in this substance were not as large as in the body of the uterus.

Upon lifting up the body of the womb to dissect it out of the pelvis, it was found to be so friable as to come off in the hand, leaving the vaginal portion of the cervix in the pelvis.

The cellular tissue was so soft in all parts as to let the fingers into it with the least possible force; but throughout its substance run thread-like bands of fibrous tissue, which had united the womb to the bladder, and woven its way to the pelvic walls. Around the rectum, it was found much more abundantly: it bound that canal to the sacrum so closely that only a finger could be introduced into its caliber from above the level of Douglas' cul-de-sac downward.

The bladder was found to contain a few ounces of *healthy looking* urine. The left kidney, with the upper portion of its ureter, contained two ounces of urine ; the right one about an ounce. The intestines were greatly distended with gas, and half liquid fæces, from the stomach downward. These walls were thin and tender, though having the appearance of being perfectly natural. The stomach was empty, and so contracted as to appear but a slight dilatation of the alimentary canal; though, like the intestines, its walls appeared natural.

When closing the incisions of the abdominal wall, it was found to be so soft as scarcely to be held between the thumb and finger, without pieces tearing away; and the force required to thrust a common suture needle through it was no more than it would require to pierce the thinnest muslin, from the pubes to umbilicus ; yet to the naked eye its appearance was perfectly natural.

The diaphragm was pushed up to the fourth rib on the right side, and on the left to the fifth intercostal space. The gall bladder was greatly distended with its natural contents. The spleen much atrophied.

The tumor was submitted to Dr. Danforth for microscopic examination.

NOTE.—Upon examining the mass left with me by Dr. Thompson, I could not make out that any distinct and isolated tumor ever existed. The mass seems to include the uterus, and its appendages; the whole united and consolidated by inflammatory proliferation of connective tissue. Microscopic examination of thin sections taken from different portions of the specimen, show essentially the same structure, namely, small spindle cells woven together in circular or elliptical groups, small round cells enclosed in the cavities formed by the spindle cells, or lying in the spaces between them, and numerous tortuous thickened blood vessels. As to the origin of the morbid growth, it seems to me impossible to decide. It may have commenced, as an intra-mural fibroid which became inflamed, and from which the inflammatory process extended to the general pelvic connective tissue, producing excessive hypertrophy and induration of that tissue, or it may have been wholly due to general inflammatory proliferation of the connective tissue cells. I am inclined to adopt the latter view, because it most satisfactorily explains and accounts for the conditions which existed. I. N. D.

Reported July 16, 1877.

